# Diarrhoeal prevalence and handwashing practices of children attending early childhood development centres in KwaZulu-Natal, South Africa

**DOI:** 10.4102/hsag.v27i0.1923

**Published:** 2022-10-25

**Authors:** Samukelisiwe N. Ntshangase, Shanaz Ghuman, Firoza Haffejee

**Affiliations:** 1 Department of Community Health Studies, Faculty of Health Sciences, Durban University of Technology, Durban, South Africa; 2 Department of Basic Medical Sciences, Faculty of Health Sciences, Durban University of Technology, Durban, South Africa

**Keywords:** diarrhoea, hygiene, preschool children, parent or guardian handwashing, teacher handwashing

## Abstract

**Background:**

Diarrhoea, a leading cause of childhood morbidity and mortality, spread through contaminated food or water or from person to person, is a major cause of hospitalisation in South African children.

**Aim:**

To determine if hygiene practices of parents or guardians and early childhood development centre (ECD) educators contributed to diarrhoea in children attending the centres.

**Setting:**

The study was conducted at ECD centres in Mpumalanga Township of KwaZulu-Natal province, South Africa.

**Methods:**

A descriptive cross-sectional study was conducted at 10 ECD centres. Parents or guardians (*n* = 385) and educators (*n* = 121) answered self-administered questionnaires. Frequencies, bivariate associations and multivariate regression modelling were conducted.

**Results:**

The prevalence of diarrhoea in children ≤ 5 years was 67.3%. Most parents or guardians washed their hands after defecating and handling a child’s faeces as well as before preparing food. Handwashing after urination was low. Washing of children’s hands after these events was lower. Although all educators reported always washing the child’s hands after defecating and before handling or eating food, they were less likely to wash the children’s hands after urination (*p* = 0.003). Childhood diarrhoea was associated with the type of toilet, households with pit latrines having a higher prevalence of diarrhoea (*p* < 0.001). It was also associated with washing of children’s hands after urination (*p* = 0.014), before handling or eating food (*p* = 0.001) and with increased number of children in the household (*p* = 0.001).

**Conclusion:**

In this population, the high prevalence of diarrhoea is related to the number of children in a household and handwashing practices.

**Contribution:**

This study highlights the importance of handwashing practices in the prevention of diarrhoea in children.

## Introduction

Diarrhoeal disease is reported by the World Health Organization (WHO) as the second leading cause of mortality in children under 5 years old; worldwide, it is responsible for the death of about 525 000 children annually (WHO 2017). Diarrhoea may be defined as the passage of three or more loose or liquid stools per day, or more frequent passage than is normal for the individual, and it can last for up to several days (WHO 2017). Because of poor hygiene practices, diarrhoea spreads through contaminated food or drinking water and from person to person. The infection occurs in the intestinal tract and is caused by a variety of bacteria, viral and parasitic organisms. Globally, studies have found diverse prevalence patterns across regions, countries or time periods (Azage et al. 2017; Fagbamigbe, Morakinyo & Abatta 2017; Oketcho et al. 2012). Research has shown that caregivers’ concern for their children’s health and action to treat the illness to the best of their knowledge and abilities is of importance (Carter et al. 2015; Cunnama & Ayako Honda 2016; Freeman et al. 2014). According to a systematic review on global handwashing, within regions in populations with similar income levels, handwashing after possible contact with excreta is not universally practised (Freeman et al. 2014). Internationally, there are nearly 1.7 billion cases of childhood diarrhoeal disease every year (WHO 2017).

The Sustainable Development Goals (SDGs), launched in 2015, aimed to end child mortality by 2030 as well as to reduce preventable deaths of newborns and children under 5 years of age (United Nations International Children’s Emergency Fund 2019). Attaining the SDG targets will prevent 11 million deaths of children under 5 years of age, but if the current trends continue, approximately 52 million children under 5 years will die before 2030 (UNICEF 2019). Efforts are needed in countries located in sub-Saharan Africa and South Asia, which are falling behind in achieving the SDGs (UNICEF 2019). In South Africa (SA), the population is approximately 55 653 654 (Statistics South Africa 2016), with the highest population aged from birth to 4 years old, among black Africans. South Africa has improved the health and well-being of children through the introduction of the rotavirus vaccine into the National Immunisation Programme in 2009 (Page et al. 2017). A study reported that this is the only known preventative measure against rotavirus diarrhoea (Mapaseka et al. 2010). Acute diarrhoea is a major cause of hospitalisation in SA, especially in children under 2 years of age (Groome & Madhi 2011). In 2000, diarrhoeal diseases contributed to 2.9% of the leading causes of morbidity in SA (Statistics South Africa 2015).

Preschools are the most populated environments for children under 5 years of age, as they provide early childhood stages of education. In 2014, approximately 50.8% of children up to the age of 4 years attended Early Childhood Development (ECD) centres in KwaZulu-Natal (KZN), SA (Department of Health 2016a). The population of KZN province in SA is approximately 11 065 240, with 1 343 532 in the age group of birth to 4 years old (Statistics South Africa 2016). Diarrhoea is the main cause of morbidity and mortality in KZN, even though case fatality decreased between 2014–2015 and 2015–2016 (Department of Health 2016b). Parents, guardians and ECD centres can prevent the development of diarrhoea in children by adopting practices such as the use of clean water, sanitation and hygiene (WASH), which have been proven to avert more than 50% of diarrhoeal deaths (Chola et al. 2015).

Programmes provided by ECD centres include community-based play groups operating for specific hours; outreach and support programmes provided at household level for young children, their families or caregivers; parenting support; enrichment programmes; and support for psychosocial needs of young children and their families (Department of Social Development [DSD] 2015). It is imperative that ECD centres are competent in reducing the risks of diarrhoea as much as possible through education and training that will extend to parents and guardians of children attending the centres.

This study aimed to assess the prevalence of diarrhoea in children aged 5 years and under at the ECD centres and to identify risk factors that may have contributed to diarrhoea in these children.

## Methods

### Study design

A descriptive cross-sectional study design was used to assess the knowledge, attitudes and practices of educators and parents or guardians regarding hygiene. The study consisted of two phases of data collection which formed part of the basis of the study. Phase 1 consisted of collecting data from parents or guardians of children attending the ECD centres through self-administered questionnaires in either English or IsiZulu. Phase 2 consisted of collecting data from ECD centre educators via self-administered structured questionnaires in either English or isiZulu. Data collection occurred in 2018 and 2019. The total number of centres registered with the DSD in the area was 41 at the time of the study, with the total number of educators approximating 177 and 3326 children attending the ECD centres.

A total number of 506 participants were recruited for the study, 385 of whom were parents or guardians and 121 were educators.

### Setting

This study was conducted in Mpumalanga Township within the parameters of the eThekwini Outer West subdistrict, KZN, SA. This area has a population of approximately 62 406 people (Statistics South Africa 2016). eThekwini district statistics for 2011–2014 revealed that this subdistrict was one of the worst affected subdistricts with diarrhoeal cases (Department of Health 2015). Early Childhood Development Centres or crèches are facilities that provide learning and support appropriate to the child’s developmental age.

### Study population and sampling strategy

The study population included all educators at the ECD centres and parents or guardians of children who were 5 years and under attending the ECD centres in the Mpumalanga Township. The educators were directly involved in the day-to-day teaching and monitoring of children in the ECD centres as well as in the preparation and provisioning of meals for the children. The study sites provided direct access to parents or guardians of the children, who formed the focus of this study. The sample size was determined using the statistical software Raosoft Sample Size Calculator. There are approximately 41 ECD centres with a total of 177 educators, and using this total population of educators, with a 95% confidence interval, and a 5% margin of error, a minimum sample size of 120 educators was calculated. Similarly, from a total of 3326 children attending the ECD centres, a minimum sample size of 384 parents and guardians of children was calculated. Simple random sampling was used in order to achieve a degree of accuracy and representativeness (Connaway & Radford 2017). The 41 ECD centres were allocated numbers 1–41, and the ballot method was utilised to select the sample. A total of 135 questionnaires were issued to educators, of which 121 (90%) questionnaires were returned. The parents or guardians were selected from the same schools that were randomly selected for the educators. The total questionnaires issued to parents or guardians were 616, of which 385 (63%) completed questionnaires were returned.

### Questionnaire development

The questionnaire for parents or guardians of children attending ECD centres had 29 questions adapted from the UNICEF Multiple Indicator Cluster Surveys (MICS) questionnaires (UNICEF 2013). Data were collected on socio-economic status of the household and adapted from MICS Questionnaire for Children Under Five (UNICEF 2017a), the MICS6 Questionnaire for Individual Women (UNICEF 2013) and MICS6 Household Questionnaire (UNICEF 2017b).

The questionnaire for ECD centre educators had 25 questions adapted from UNICEF MICS questionnaires and the Perception Survey for Health-Care Workers (WHO 2009). Data on socio-economic status of the ECD centres were adapted from MICS6 Questionnaire for Children Under Five (UNICEF 2017a) and MICS Household Questionnaire. This questionnaire evaluated perceptions of ECD educators and was derived from the Perception Survey for Health-Care Workers survey (WHO 2009).

Both questionnaires were translated into isiZulu by the primary investigator (PI), who is fluent in both English and isiZulu. For verification, the isiZulu version was subsequently back-translated into English by an independent translator. Similarly, the letter of information and consent forms were also translated and confirmed as correct.

Validity and reliability were ensured through a focus group discussion, which thoroughly interrogated both questionnaires, as well as a subsequent pilot study. The focus group discussion was held by a team of five members, comprising three researchers and two academics who were not involved in the research study. Following this discussion, some amendments were made regarding the layout of the questionnaire, as well as some rephrasing to ensure understanding of all questions within the local context. A pilot study subsequently ensued. In total eight parents or guardians participated in the pilot study, two of whom answered the questionnaire in isiZulu and six in English. None of them experienced any difficulties in understanding any questions nor in answering the questionnaire. Seven ECD centre educators also participated in the pilot study, five of whom answered the isiZulu version, and two educators answered the English version. The participants found the questions easy to understand and no changes were made. All pilot study participants were excluded from the main study.

### Data collection

The study was presented to the ECD centre principals and managers during their monthly meeting where all ECD centres in Mpumalanga Township meet with other stakeholders. The ECD centres were informed of the aim and objectives of the research and of the researcher’s forthcoming sampling procedures. Prospective participants who met the inclusion criteria (ECD centre educators and parents or guardians of children under 5 years attending the centres) were all given a letter of information and consent form. After receiving signed informed consent, parents or guardians of children under 5 years attending the ECD centres were given a self-administered questionnaire to fill out. The questionnaire was in their language of choice (either English or isiZulu). Those parents or guardians who were unable to read or write were asked to request assistance from another member of their household, who read out the questions and scribed the answers verbatim. Those ECD centre educators who provided signed informed consent were also issued with a questionnaire in their language of choice (either English or isiZulu). Participants were informed to return the questionnaires to the ECD centres and place them in a sealed collection box which was left at each centre. Completed questionnaires were collected after a period of two weeks.

The parents’ questionnaires were placed in the children’s school bag for parents or guardians to fill and return to the school. The educators checked each child’s bag daily for returned questionnaires and placed them in the collection box issued to each ECD centre for a month. Because of the poor response from parents or guardians with this technique, the first author subsequently administered the questionnaires personally to parents or guardians during parents’ meetings at the end of the term.

### Data analysis

Statistical analysis was performed using SPSS version 26.0. Frequency distribution of categorical variables and means, standard deviation and ranges of continuous variables were calculated. Pearson’s chi-square test was used where applicable to determine associations between categorical variables. Bivariate and multivariate regression modelling was done with the inclusion of relevant covariates. Odds ratios (ORs) were calculated for binary outcome variables. When the dependent variable was numerical and the independent variable(s) were nominal, an effect size was determined using a partial eta-squared value. *P*-values < 0.05 were considered statistically significant.

### Ethical considerations

Ethical approval to conduct the study was obtained from the Durban University of Technology, Institutional Research Ethics Committee (reference number: REC 133/17). Gatekeeper permission was obtained from the DSD. Participation was voluntary and informed consent was obtained from all the participants; no names were used on the questionnaires which were collected separately from the consent forms. All collected data were coded, while personal details were not recorded. Data were securely stored in a locked cupboard accessible only to the researcher, and the records will be kept for a period of 5 years before being shredded and disposed of appropriately. All electronic data were stored on a password-protected file and will be deleted after 5 years.

## Results

A total of 121 educators and 385 parents or guardians from 10 out of 41 ECD centres in Mpumalanga Township formed part of the study.

### Participant demographics

As shown in Table 1, the ratio of men to women was approximately 1:9, with the majority being single. All other parental demographic characteristics are presented in Table 1.

**TABLE 1:** Demographic characteristics of parents or guardians (*n* = 385).

Demographic characteristic	Frequency (*n*)	%
**Gender**		
Male	39	10.1
Female	346	89.9
**Marital status**		
Single	282	73.4
Married	81	21.1
Widowed	20	4.9
Divorced	2	0.5
**Education level**		
None	13	3.4
Primary school	40	10.5
Secondary school	210	54.6
Tertiary	122	31.5
**Occupation**		
Employed	170	44.7
Unemployed	201	52.5
Pensioner	12	3.1

The mean age of parents or guardians was 33.2 ± 11.0 years (range: 16–71 years). The educators had an average of 4 years’ experience teaching at ECD centres (4.6 ± 2.77 years). The majority of educators only had a senior high school education (*n* = 59, 59.6%), and 23.2% (*n* = 23) had a National Certificate in ECD, while 4% (*n* = 4) of educators had not completed their high school education.

The mean number of people per household was 5.7 ± 2.2, with an average of two children per household (1.8 ± 1.3). Most children were aged between 3 and 5 years, as shown in Table 2.

**TABLE 2:** Age distribution of children.

Age	Children in household		Children in ECD centre	
	Frequency	%	Frequency	%
0–6 months	77	13.6	27	7.9
7–18 months	82	14.4	76	22.2
19 months to < 3 years	147	25.9	84	24.4
1. 3–5 years	262	46.1	156	45.5

**Total**	**568**	**100**	**343**	**100**

ECD, early childhood development.

### Water and sanitation

The majority of households (80.8%) had an indoor tap (Table 3). The type of toilets differed among the households (*p* < 0.001). The majority of households had an indoor toilet (*n* = 333, 86.5%), 45 households (11.7%) had toilets within the yard and only four households (1%) had to use a community toilet outside the yard. Of these toilets, 350 (91.7%) had a flushing system, 30 were pit latrines (7.8%) and two (0.5%) were portable public toilets. Most of the toilets (83.9%) had a sink next to it. Only five respondents (1.3%) stated that there was no sink, but they used a dish to wash their hands (Table 3).

**TABLE 3:** Household source of water.

Variable	Frequency	%
**Source of water**		
Indoor tap	311	80.8
Outdoor tap on premises	70	18.2
Public tap: 500 m – 1 km	2	0.5
Public tap: > 1 km away	1	0.3
Tanker-truck, vendor	1	0.3
**Position of tap in relation to toilet**		
Next to the toilet	323	83.9
Outside toilet but in the dwelling	33	8.6
Within the yard	16	4.2
No tap; used a dish	5	1.3

Seven ECD centres had indoor taps and indoor toilets. The other three had these facilities outside the centre. All ECD centres had flushing toilets.

### Hygiene practices of parents or guardians

**FIGURE 1:**
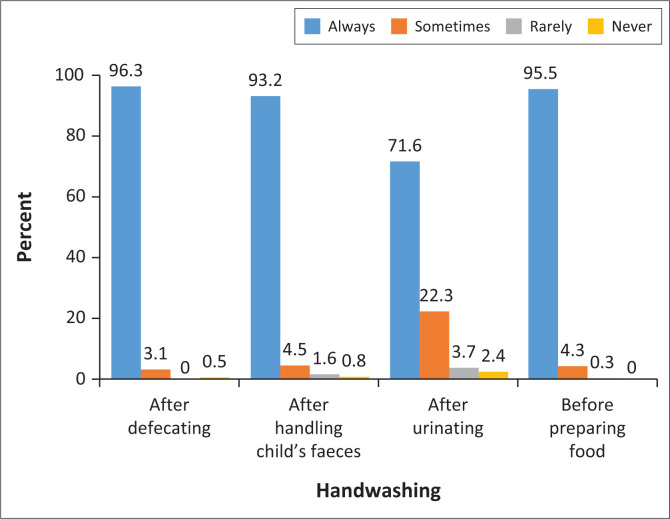
Handwashing practices of parents or guardians.

Figure 1 shows the handwashing practices of the parents or guardians. Most of them always washed their hands after defecating and after handling a child’s faeces, as well as before preparing food.

The frequency of handwashing after defecating was associated with the source of water, as handwashing decreased when the water source was further away from the house (chi-square tests; *p* < 0.001). Those respondents who had an indoor tap (*n* = 307, 85%) washed their hands more often after defecating than did those with an outdoor tap on the premises (*n* = 51, 14.1%) and those who used a public tap (*n* = 3, 0.8%). The frequency of handwashing was inversely related to the distance of the water source (*p* = 0.012). However, handwashing of parents or guardians after urination was not associated with the source of drinking water (*p* = 0.094), the distance of the water source (*p* = 0.061) or the distance of handwashing sink from the toilet (*p* = 0.864).

Similarly, more parents or guardians washed their hands after handling a child’s faeces when the water source was indoors and less frequently when the source of water was further away from the household (*p* = 0.011). Parents or guardians were less likely to wash their hands after assisting children who went to the toilet on their own compared to a child who used nappies; this was, however, not significant (*p* = 0.065).

However, there was a correlation between parents’ or guardians’ handwashing frequency before preparing food and the location of the handwashing sink (*p* = 0.010), as their handwashing was more frequent before preparing food when there was an indoor tap (*n* = 296, 84.3%) compared to an outdoor tap on the premises (*n* = 52, 14.8%) or a public tap (*n* = 3, 0.9%).

Many of the respondents (*n* = 280, 74.7%) stated that they always used soap and water when they washed their hands, with approximately one-fifth (*n* = 72, 19.2%) indicating that they used water only (*p* < 0.001). Many respondents experienced challenges related to the availability of soap (*n* = 64, 63.4%) and inadequate water supply (*n* = 23, 22.8%).

### Handwashing of children by parent or guardian

**FIGURE 2:**
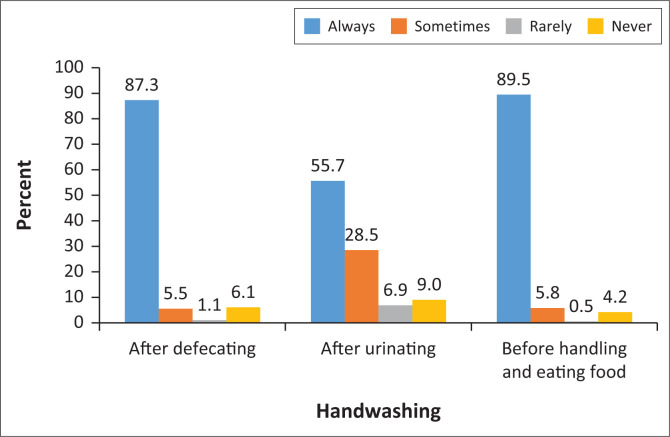
Frequency of handwashing after specific activities.

Figure 2 depicts washing of children’s hands by the parents or guardians. The majority of parents or guardians reported that they always washed their children’s hands after the child had defecated (*n* = 331, 87.3%) and before the child ate (*n* = 340, 89.5%). However, only half of the respondents reported always washing their child’s hands after urination (*n* = 211, 55.7%).

### Hygiene practices of early childhood development educators

The majority of educators reported having received formal training on hand hygiene (*n* = 115, 95%), while 5% did not receive any formal training. Hand hygiene training (*n* = 115, 95%) was predominantly provided by the Department of Health and 0.8% (*n* = 1) reported having received training from the DSD.

All respondents reported that the ECD centre provided and prepared meals for children. Three-quarters (76.9%) of the respondents stated that the ECD centre prepared two meals and 23.1% (*n* = 28) provided more than two meals. Most of the ECD centres sourced food items from their own food garden initiatives.

The majority of respondents (*n* = 110, 90.9%) stated that hand hygiene was of very high importance in their ECD centre. All the respondents indicated that children were taught the importance of handwashing, and the children always used soap and water when washing hands. There was no association between ECD educators’ level of training and children being taught the importance of handwashing (*p* = 1.000). All respondents reported always washing the child’s hands after defecating and before handling or eating food. Most parents or guardians (*n* = 115, 95.9%) washed their child’s hands after playing outside in the sand, and 84.2% (*n* = 101) washed the child’s hands after urination. Educators were less likely to wash the children’s hands after urination than any other activity (*p* = 0.003).

The majority of educators (*n* = 81, 66.9%) stated that inadequate water supply was a major challenge for handwashing, followed by a combination of inadequate water supply and unavailability of soap (*n* = 15, 12.4%). Ten (8.3%) respondents reported that children could not wash their hands properly and hence required assistance.

### Prevalence of diarrhoea episodes in children under the age of 5 years

A total of 231 (67.3%) ECD children under the age of 5 years had diarrhoea in the 12 months preceding the study. The effect size of the total number of people within the household on diarrhoea was low (partial eta-squared value, *η*^2^ = 0.002, *p* = 0.389). This implies that there was little or no effect of the total number of people in the house on the child having diarrhoea. However, there was a small to medium effect of the number of children in the house on a child having diarrhoea (partial eta-squared value, *η*^2^ = 0.036, *p* < 0.001), showing that children are more likely to get diarrhoea from other children, compared to adults. There was little to no effect of the location of the toilet on the child having diarrhoea (partial eta-squared value; *η*^2^ *=* 0.009, *p* < 0.001) and a small to medium effect of the type of household toilet (partial eta-squared value; *η*^2^ *=* 0.062, *p* < 0.001).

The prevalence of childhood diarrhoea was associated with washing of a child’s hands after urination (*p* = 0.014) and before handling or eating food (*p* = 0.001) but was not associated with washing the child’s hands after defecating (*p* = 0.697). Similarly, there was an association between childhood diarrhoea and the parents or guardians washing their own hands after urination (*p* = 0.05), before handling food (*p* = 0.020), as well as after defecating (*p* = 0.033).

More than half of the ECD educators (*n* = 73, 61.9%) indicated that a child had gone to school while having diarrhoea. Children were less likely to go to school with diarrhoea when the ECD centre provided two meals a day compared to those that provided more than two meals a day (OR = 0.288, 95% confidence interval [CI] = 0.116–0.711, *p* = 0.011), but diarrhoea was not associated with the source of water at the centre (*p* = 0.369). The majority of respondents (*n* = 118, 97.5%) reported that children were assisted in the ECD centres when going to the toilet.

## Discussion

This study showed a high prevalence of childhood diarrhoea among attendees of ECD centres in a rural district of KZN, SA. Diarrhoeal prevalence was associated with the number of children in the household and handwashing practices of children as well as their parents or guardians.

The WHO estimated global childhood diarrhoeal disease cases to be nearly 1.7 billion per annum (WHO 2017), and that in KZN, diarrhoea was one of the main causes of morbidity and mortality in children under the age of 5 years (Department of Health 2016a). These estimates were translated to the outcome of this study, where two-thirds of children had had diarrhoea at some point 12 months prior to enrolling in this study.

Although the total number of people living in the household did not affect the incidence of diarrhoea, the incidence was higher in those homes that had more children under the age of 5 years. Numerous studies have reported similar findings regarding the presence of young siblings in the household. Studies in India and Ethiopia reported that households with siblings under the age of 5 years had a higher incidence and duration of diarrhoea (Godana & Mengistie 2013; Kattula et al. 2015). Being a younger sibling also increased the risk of diarrhoea, independent of attendance at preschools (Gudnason et al. 2012). An increase in the number of children in a family results in overcrowding, which adversely affects hygiene conditions and increases the chance of contact with pathogens when the children interact (Godana & Mengistie 2013). The mother might also be overwhelmed by the little children competing for attention (Godana & Mengistie 2013), thereby neglecting to practise adequate hygiene.

Washing of hands at critical moments, such as after defecating and urinating as well as before handling or eating food, is important. We report an association between childhood diarrhoeal prevalence and washing of both parents’ and children’s hands at these times. Although the majority of parents or guardians washed their own hands after defecating, after handling a child’s stool and before preparing food, washing of the child’s hands at these critical moments was lower by parents or guardians, but ECD educators were consistent in washing the child’s hands at these times. In contrast, the frequency of parental handwashing, as well as washing of a child’s hands after urination by both parents or guardians and educators, was low. The main purpose of washing hands is to cleanse the hands of pathogens and chemicals which can cause personal harm or disease. Handwashing with soap removes transient potentially pathogenic organisms from hands and it is not sufficient to wash hands with only water after critical events like defecation. If individuals wash their hands, they are less likely to transmit pathogens via the hand–mouth route. The majority of parents or guardians reported washing hands only with water, possibly because of the challenges they experienced in obtaining soap. The researchers recognise that they hail from a low socio-economic background; hence, limited funds are not prioritised for the purchase of items such as soap. A study in the Eastern Cape province of SA also reported that soap was not used for handwashing after defecating (Demberere et al. 2016). Washing hands with soap and water is effective in preventing diseases because the soap breaks down grease and dirt that carry pathogens (Demberere et al. 2016). A study in Kenya found that the presence of soap in the household was associated with fewer days of diarrhoea (Kamm et al. 2014). Similarly, another study found that children whose mothers did not practise handwashing with soap at critical times were more likely to develop diarrhoea (Gebru, Taha & Kassahun 2014).

The low frequency of handwashing after assisting a child in the toilet, and after changing the child’s nappies, corroborates with that of a study conducted in the Eastern Cape province, which was associated with the lack of handwashing facilities next to the toilet (Demberere et al. 2016). Similarly, a study in Nigeria observed that mothers almost never washed their hands after cleaning a child’s bottom (Dairo, Ibrahim & Salawu 2017). Furthermore, many parents or guardians whose children used nappies disposed the stools in the municipal collected waste and some left it out in the open. Children whose stools were disposed of unsafely were more likely to suffer from diarrhoea than children whose stools were disposed of safely (Bawankule et al. 2017; Demberere et al. 2016). Another study in India found that the lack of toilets resulted in unsafe disposal of stool, thereby increasing the risk of exposure to diarrhoea-causing pathogens (Bawankule et al. 2017).

The present authors also found that childhood diarrhoea was associated with the type of toilet, those households with pit latrines having a higher prevalence of diarrhoea compared to those with flushing toilets. High incidence of diarrhoea was associated with defecation in open, in bushes and near the river banks (Demberere et al. 2016). A similar study in Ethiopia found that children from homes without any type of toilet and defecating in the open field were more likely to have diarrhoea than children whose families had a toilet (Gebru et al. 2014).

Water was easily accessible in this study population. The majority of households and all ECD centres had indoor taps. A long time taken to collect water compromised hygiene practices, resulting in high diarrhoeal incidence because when it took time to collect water, it was used sparingly, with handwashing facilities being considered a waste of water (Demberere et al. 2016). This study indicated that parents or guardians washed their hands more frequently when the source of water was on the premises rather than further away from the house. When handwashing sinks were closer to the toilet, the frequency of handwashing was higher compared to when handwashing sinks were further away (Biran et al. 2009).

The ECD centre educators indicated that more than 60% of children attended school while having diarrhoea. It was noted that most of these were at schools that provided more than two meals per day. These children are from an under-resourced setting, with over half of the parents being unemployed. It is thus highly likely that the children are sent to school despite their illness, as going to school meant that they would be getting their meals for the day, something that the parents possibly could not afford. The ECD policy states that ECD staff members ought to be trained about the methods of transmission and prevention of illness (DSD 2015). Parents need to be notified and the child should be isolated from other children (DSD 2015). However, the latter is not happening with diarrhoea and other illnesses.

## Limitations

This study was conducted at ECD centres within one district in KZN province, SA. Therefore, the results cannot be generalised to the entire province. Larger studies in various semi-urban, rural and semi-rural districts are required to gain a better understanding of the risk factors of childhood diarrhoea in SA.

## Conclusion

This study concludes that in this population, the high prevalence of diarrhoea is related to the number of children in a household, handwashing practices and the availability of soap. Training of parents or guardians is required to ensure that proper hygiene practices are implemented at home. Early childhood development centres could play a role in having training sessions for parents or guardians of children who are enrolled at the ECD centres. Education of ECD staff members and parents or guardians is warranted to increase handwashing practices after urination, as pathogens may spread from the toilet or via the anus–hand–mouth route during this time. Ultimately, this will reduce under-five child mortality, keeping in line with SDG 3 (United Nations 2015).
